# *Streptomyces songxianensis* sp. nov. SX92^T^: biocontrol of tobacco black shank and modulation of the rhizosphere microbiome

**DOI:** 10.3389/fmicb.2026.1820235

**Published:** 2026-08-25

**Authors:** Mengyu Zhang, Lei He, Chengjun Li, Shuhe Wang, Jianqiang Xu, Zhengxiong Song, Yebin Kang

**Affiliations:** 1 College of Agriculture, Henan University of Science and Technology, Luoyang, Henan, China; 2 Henan Branch of China National Tobacco Corporation, Zhengzhou, China; 3 Luoyang Branch of Henan Tobacco Company, Luoyang, China; 4 Tobacco Research Institute, Henan Academy of Agricultural Sciences, Xuchang, Henan, China; 5 College of Horticulture and Plant Conservation, Henan University of Science and Technology, Luoyang, Henan, China

**Keywords:** biocontrol, genome analysis, microbial diversity, plant disease suppression, *Streptomyces*, tobacco

## Abstract

*Streptomyces* species are well-known for their potential in biocontrol and plant growth promotion, with the rhizosphere serving a rich reservoir for novel isolates. In this study, a *Streptomyces* strain (SX92^T^) was isolated from the rhizosphere of healthy tobacco plants. In dual-culture assays, SX92^T^ displayed broad-spectrum antagonistic activity against six major fungal pathogens of tobacco, with the highest inhibition (59.22%) against *Phytophthora nicotianae*, the causal agent of tobacco black shank. Polyphasic taxonomic characterization, combining 16S rRNA gene phylogeny, distinctive physiological traits, chemotaxonomic markers (LL-diaminopimelic acid, major menaquinones MK-10(H₄) and MK-9(H₈), and predominant fatty acids anteiso-C₁₅:₀ and C₁₆:₀), and genome-based metrics (ANI and dDDH), clearly distinguished SX92^T^ from its closest relatives. Accordingly, strain SX92^T^ is proposed as the type strain of a novel species, *Streptomyces songxianensis* sp. nov. The genome of SX92^T^ is 9.69 Mb in size with a G + C content of 71% and contains 26 biosynthetic gene clusters, including one showing 100% similarity to the albaflavenone cluster. In field trials, application of SX92^T^ fermentation broth significantly improved tobacco agronomic traits and reduced black shank incidence by 44.97%. Furthermore, SX92^T^ treatment reshaped the rhizosphere microbiome by enriching beneficial bacteria such as *Flavobacterium* and altering the relative abundance of specific fungi, including a reduction in the arbuscular mycorrhizal fungus *Rhizophagus irregularis*. It also shifted soil enzyme activities, with increased cellulase and decreased catalase levels. These findings establish *Streptomyces songxianensis* SX92^T^ as a promising multifunctional biocontrol agent for sustainable tobacco production.

## 1 Introduction

Plant diseases pose a significant biotic constraint to global agricultural sustainability. Among these, soil-borne diseases are particularly challenging to manage due to their high recurrence rates and are difficult to control (Meshram and Adhikari, 2024). Tobacco black shank disease, caused by the oomycete pathogen *Phytophthora nicotianae*, is a devastating soil-borne disease that can cause yield losses of 30–50% in tobacco, severely threatening the sustainable development of the tobacco industry (Zhu et al., 2024). Current management strategies rely heavily on chemical fungicides such as metalaxyl and dimethomorph. However, the long-term use of these chemicals not only leads to widespread pathogen resistance but also disrupts the soil microbial balance and exacerbates environmental pollution (Guo et al., 2020; Han et al., 2016). Consequently, biological control agents (BCAs), especially those derived from beneficial microorganisms, have emerged as a promising alternative for sustainable agriculture.

*Streptomyces* spp. are particularly compelling candidates for BCAs, renowned for their exceptional metabolic diversity and unparalleled ability to produce antimicrobial secondary metabolites (De Troyer et al., 2025; Kotowska et al., 2024; Lahlali et al., 2022). Taxonomically, initial classification of the *Streptomyces* members was primarily based on phenotypic characteristics such as colony and spore morphology, and physiological traits. Currently, the taxonomy of *Streptomyces* relies on a polyphasic approach combining phenotypic, chemotaxonomic, and genotypic characteristics. Typical descriptions include morphological features (aerial mycelium and spore morphology), chemotaxonomic markers (cell wall composition, polar lipids, menaquinones), and physiological and biochemical properties. Modern taxonomic criteria further integrate genomic standards, such as Average Nucleotide Identity analysis and digital DNA–DNA hybridization based on whole-genome sequences, which provide reproducible and objective means for species demarcation (Komaki, 2023). Many streptomycetes exhibit direct antagonism against a broad range of plant pathogens. Furthermore, certain strains demonstrate plant growth-promoting (PGP) traits, offering dual benefits for crop production (He et al., 2024; Peng et al., 2020; Qi et al., 2021).

For instance, *Streptomyces exfoliates*. FT05W and *Streptomyces cyaneus*. ZEA17I have been reported to demonstrate antagonistic activity against *Phytophthora parasitica* var. *nicotianae*, the causal agent of tobacco black shank, with inhibition rates of 68.82 and 76.18%, respectively(Chen et al., 2017). *Streptomyces albidoflavus.* S20 has been described as a highly promising multifunctional biocontrol agent, effectively managing tobacco brown spot through both direct antagonism and growth promotion, representing a sustainable and efficient biological control strategy(Wang et al., 2025). Nevertheless, translation promising *in vitro* results into consistent field efficacy remains a major challenge. Numerous biocontrol candidates fail under complex field conditions due to poor environmental adaptability, inconsistent disease suppression, or an inability to integrate synergistically within the resident rhizosphere microbiome (Devi et al., 2022; Pirttilä et al., 2021; Xun et al., 2021; You et al., 2025). Critically, the impact of introduced BCAs on the structure and function of the resident soil microbiota, which are essential determinants of plant health and soil ecosystem stability, is often remains poorly understood (Bonanomi et al., 2018; Wang et al., 2025).

Despite the recognized potential of *Streptomyces* as BCAs, several critical knowledge gaps hinder their optimized application. First, although laboratory screening identifies numerous antagonistic strains, rigorous validation of their disease control and plant growth efficacy under field conditions is essential for assessing practical utility and remains a pivotal step for many candidates (Carlucci et al., 2023; Shi et al., 2024). Second, the success and persistence of a BCA depend fundamentally on its physiological resilience and metabolic adaptability, enabling survival and activity within the specific physicochemical conditions of the target rhizosphere (Dow et al., 2023). Comprehensive assessment of growth conditions, stress tolerance, and substrate utilization capabilities is therefore critical for predicting ecological fitness, yet such data are often incomplete (Ge et al., 2024). Moreover, precise taxonomic identification is essential for accurate strain characterization, establishing novelty, ensuring regulatory compliance, and linking functional traits to species identity (Ryabova and Gagarina, 2022). This requires a polyphasic approach integrating whole-genome sequencing with traditional chemotaxonomic and phenotypic analyses. Finally, and most importantly, a thorough understanding of the ecological impact of introducing *Streptomyces* BCAs into the rhizosphere, particularly their effects on the diversity, composition, and functional dynamics of native bacterial, archaeal, and fungal communities, is crucial for evaluating their holistic biocontrol mechanisms (Kumar et al., 2022; Nazari et al., 2023).

An ideal BCA should not only directly suppress pathogens and enhance plant growth but also positively modulate the rhizosphere microbiome toward a beneficial and resilient state, thereby improving overall soil health (Moussa and Iasur Kruh, 2025). Strains that combine this triad of functions-pathogen antagonism, plant growth promotion, and microbiome engineering-represent a highly desirable yet underexplored resource for sustainable agriculture.

In this study, we isolated and characterized a novel *Streptomyces* strain, SX92^T^, from tobacco rhizosphere soil. Polyphasic taxonomic analysis confirmed its identity as a new species, for which we propose the name *Streptomyces songxianensis* sp. nov. The strain exhibits broad-spectrum antagonistic activity against major tobacco pathogens, supported by genomic analysis revealing abundant secondary metabolite biosynthetic gene clusters. Field trials demonstrated its ability to enhance tobacco growth and significantly reduce the incidence of black shank disease. Moreover, SX92^T^ altered soil enzyme activities and reshaped the microbial community structure by enriching beneficial taxa while reducing pathogen abundance. These findings collectively indicate that SX92^T^ is a multifaceted biocontrol agent with strong potential for sustainable tobacco production.

## 2 Materials and methods

### 2.1 Microbial strains

Strain SX92^T^ was isolated from rhizosphere soil of tobacco plants in Luoyang, Henan Province, China (34°39′N, 112°24′E). This strain has been deposited in the Guangdong Microbial Culture Collection Center (GDMCC) under accession number GDMCC 4.452 and in the Japan Collection of Microorganisms (JCM) as JCM 37854. Its complete genome sequence is available in NCBI GenBank database under accession number CP132593. The 16S rRNA gene sequence has been deposited in GenBank under accession number MH265970.1. The reference strain *Streptomyces caeruleatus* NRRL B-24802^T^ was obtained from the Leibniz Institute DSMZ-German Collection of Microorganisms and Cell Cultures and cultured under identical conditions. Pathogenic fungal strains (*Phytophthora nicotianae*, *Rhizoctonia solani*, *Alternaria alternata*, *Fusarium oxysporum*, *Botryosphaeria dothidea* and *Fusarium solani*) were provided by the Laboratory of Molecular Identification and Green Prevention and Control of Plant Diseases within the Henan province, China.

### 2.2 Culture media

Various culture media were employed for different experimental purposes: Gauze’s Medium No.1 (GM1) and its liquid counterpart, Gauze’s fermentation broth, served as the basal media for strain purification, solid-state culture, and liquid fermentation, respectively. The International *Streptomyces* Project (ISP) recommended media (ISP1 through ISP7) were used to elicit morphological diversity (including aerial hyphae, spore chains, and diffusible pigments) for observation and preliminary taxonomic identification; Nutrient Agar (NA) and Potato Dextrose Agar (PDA) were utilized to observe specific growth characteristics. GM1 contained (per liter): soluble starch, 20.0 g; KNO₃, 1.0 g; MgSO₄·7H₂O, 0.5 g; NaCl, 0.5 g, FeSO₄·7H₂O, 0.01 g, K₂HPO₄, 0.5 g; agar, 15.0–20.0 g; pH adjusted to 7.2–7.4 with 0.2 mol/L NaOH. ISP1 - ISP7 media were prepared as described by Shirling and Gottlieb (1966). NA contained (per liter): peptone, 5.0 g; NaCl, 5.0 g; beef extract, 3.0 g; agar, 15.0 g. PDA was prepared by boiling 200 g of fresh potatoes in water for 30 min, filtering, adding 20.0 g glucose and 15.0–20.0 g agar, and bringing the volume to 1 L with distilled water. All media were sterilized by autoclaving at 121 °C for 15 min and stored at 4 °C until use.

### 2.3 *In vitro* antifungal activity assay

The antifungal activity of *Streptomyces* sp. SX92ᵀ was evaluated against six tobacco pathogenic fungi (*P. nicotianae*, *R. solani*, *A. alternata*, *F. oxysporum*, *B. dothidea*, and *F. solani*) using a modified dual-culture plate method (Yang et al., 2024). An 8 mm mycelial plug of each pathogen was placed at the center of a 90 mm PDA plate. A streak of SX92ᵀ was inoculated 2.5 cm away. Plates were incubated at 25 °C, and mycelial growth was monitored daily. Controls consisted of pathogens grown alone. The experiment included three replicates per treatment. Inhibition rates were calculated when the mycelium of the control reached the plate edge using the formula:


$$\text{Inhibition rate}\;(\%)=[(R_{0}–R)/(R_{0}–8)]\times 100\%.$$

Where *R_0_* and *R* are the colony diameters (mm) in the control and treatment groups, respectively.

### 2.4 16S rRNA gene analysis and whole-genome sequencing

SX92^T^ was cultured in GM1 broth for 9 days at 28 °C (Tamreihao et al., 2018). Genomic DNA was extracted using a commercial kit (Omega Bio-Tek Co., Ltd., Guangzhou, China). The 16S rRNA gene was amplified via PCR with the universal primers 27F and 1492R. The resulting PCR product was purified and subsequently to bidirectional Sanger sequencing by a commercial service.

The obtained 16S rRNA gene sequences of SX92^T^ was compared with the sequences of type strains retrieved from the EzBioCloud database^1^ using the BLAST program to identify its nearest phylogenetic neighbours and calculate 16S rRNA gene sequence similarity. Phylogenetic trees were reconstructed using the neighbour-joining, maximum-likelihood and maximum-parsimony method in MEGA 7, following multiple sequence alignment with Clustal W. The robustness of the phylogenetic trees was evaluated using the bootstrap resampling method (1,000 replicates). (Saitou and Nei, 1987; Felsenstein, 1985; Jukes and Cantor, 1969; Kumar et al., 2016).

Whole-genome sequencing was performed commercially by Shanghai Majorbio Bio-pharm Technology Co., Ltd. using the PacBio RSII and Illumina HiSeq X-ten platforms. The complete genome was *de novo* assembled using Canu v2.1 with the PacBio reads and further polished with Illumina reads using Pilon v1.23. The sequencing coverage was approximately 100 × for the PacBio platform and 50 × for the Illumina platform. The final assembly resulted in a single contig (the chromosome) with an N50 value of 9,691,730 bp. The complete genome was assembled using SMRT analysis. Secondary metabolite biosynthetic gene clusters were identified using antiSMASH 4.0.2, and CAZymes were annotated using the CAZy database (Blin et al., 2021). The genomic G + C content was calculated from the assembled sequence (Yoon et al., 2017).

Digital DNA–DNA hybridization (dDDH) and average nucleotide identity (ANI) were calculated using the GGDC web server and ANI Calculator, respectively (Meier-Kolthoff et al., 2013; Yoon et al., 2017).

### 2.5 Morphological, cultural and physiological characterization

Mycelia grown on ISP2 agar at 28 °C for 14 days were processed for scanning electron microscopy (SEM): fixed with 2.5% glutaraldehyde, rinsed with phosphate buffer, post-fixed with osmium tetroxide, dehydrated through an ethanol series, critical-point dried, sputter-coated with platinum, and observed under SEM. Cultural characteristic and pigment production were recorded on ISP1–7 (Shirling and Gottlieb, 1966), PDA, GM1 and NA after incubation for 14 days at 28 °C (Williams and Davies, 1967). Colors were determined using Ridgway standards (Ridgway, 1912). Growth was assessed at temperatures from 5 to 40 °C (at 5 °C intervals) on GM1, at pH values from 2 to 12 (in increments of 1.0 unit) in ISP2 broth (Xu et al., 2005), and with 0–3% (w/v) NaCl in ISP2 broth. Physiological traits and carbon source utilization were tested as described by Williams et al. (1983). Tests for catalase production, milk peptonization and coagulation, gelatin liquefaction, starch hydrolysis, cellulose decomposition, and H₂S production were performed (Kamlage, 1996). All physiological tests of strains SX92^T^ and *Streptomyces caeruleatus* NRRL B-24802^T^ were repeated three times.

### 2.6 Chemotaxonomic analyses

The strain was cultured in ISP2 medium at 28 °C with shaking at 180 rpm for 7 days to obtain sufficient biomass. Cells were harvested by centrifugation (10,000 × g, 10 min) and washed twice with sterile distilled water. The washed cells were frozen at −80 °C and lyophilized to obtain dried cell powder for subsequent analyses. Diaminopimelic acid (DAP) isomer and whole- cell sugars were analysed as described by Hasegawa et al. (1983) and Lechevalier et al. (1977). Menaquinones were extracted and analyzed by HPLC (Agilent 1,200; Zorbax Eclipse XDB-C18 column; 250 × 4.6 mm; 5 μm) using methanol–isopropanol (2:1, v/v) at 1 mL/min, 40 °C, with detection at 270 nm (Minnikin et al., 1984).

Polar lipids were separated via two-dimensional TLC on silica gel plates using chloroform-methanol–water (65:25:4, v/v/v) and chloroform-methanol-acetic acid-water (80:18:12:5, v/v/v/v) (Cornatzer, 1974). Fatty acid methyl esters were analyzed by GC (Agilent 6,890) using the MIDI system (Buyer and Sasser, 2012).

### 2.7 Plant growth promotion and disease control in field trials

Field experiments were conducted in Yiyang County, Luoyang City (34°32′N, 111°46′E, altitude 413 m) from May to August 2024 using tobacco variety Zhongyan 100. Two adjacent plots with a documented history of black shank from the previous season were selected. Pre-experiment soil analysis confirmed no significant differences in key physicochemical properties, including pH and organic matter content, between the two plots. Treatments were: (1) CK: water control; (2) GM1: GM1 fermentation broth control; (3) SX92ᵀ: SX92ᵀ culture broth (1 × 10^8^ CFU·mL^−1^). The culture broth was prepared by inoculating a single colony of SX92^T^ into Gauze’s fermentation broth (GM1 without agar) and incubating at 28 °C with shaking at 180 rpm for 7 days. Cells were harvested by centrifugation (10,000 × g, 10 min), and the pellet was resuspended and adjusted to the desired concentration in sterile distilled water. Each plant received 10 mL at transplanting and again 7 days later. Each treatment included 30 plants with five replicates arranged in a randomized block design (row spacing: 110 cm; plant spacing: 55 cm). Plant height, stem circumference, leaf area, chlorophyll, and nitrogen content were measured at rosette and vigorous growth stages according to the Chinese Tobacco Industry Standard YC/T 142–2010. Disease severity was assessed at harvest according to GB/T 23222-2008 using a 0–9 scale (Gao et al., 2015). Disease index and control efficacy were calculated as follows:


$$\begin{array}{l} \text{Disease index}=[\Sigma \;(\begin{array}{l} \text{Number of diseased plants or}\;\\ \text{leaves}\;\mathrm{at}\;\text{each level}\times \text{Disease level value} \end{array})]\\ /(\begin{array}{l} \text{Total number of plants or leaves surveyed}\\ \times \text{Highest disease level value} \end{array})\times 100. \end{array}$$

$$\text{Control efficacy}\;(\%)=[\begin{array}{l} \left(\begin{array}{l} \text{Disease index of control}\\ –\text{Disease index of treatment} \end{array}\right)\\ /\text{Disease index of control} \end{array}]\times 100.$$

### 2.8 Rhizosphere soil sampling and processing

On August 23, 2024, six plants per treatment were randomly uprooted. Rhizosphere soil (1–2 mm from roots) was collected with brushes, composited from two plants per sample, yielding six composites per group. Samples were sieved (2 mm), and aliquots were stored at 4 °C for soil enzyme activity analysis (within 24 h) or immediately flash-frozen in liquid nitrogen and stored at −80 °C for microbial DNA extraction. Three samples per group were used for DNA sequencing.

#### 2.8.1 Soil enzyme activity assays

For enzyme activity assays, 5 g of fresh soil was homogenized with 45 mL of sterile phosphate buffer (50 mM, pH 7.0) and shaken at 200 rpm for 30 min. The homogenate was centrifuged at 5,000 × g for 10 min at 4 °C, and the supernatant was used as the crude enzyme extract for subsequent assays. Soil enzyme activities were determined using standard colorimetric methods. Sucrase, urease, phosphatase, and cellulase activities were measured after 24 h of incubation at 37 °C with their respective substrates, followed by centrifugation and reaction with specific reagents: DNS for sucrase and cellulase (absorbance at 540 nm), phenol-hypochlorite for urease (578 nm), and chlorinated dibromoquinoneimine for phosphatase (570 nm). Catalase activity was assessed by incubating soil with H₂O₂ for 20 min and measuring absorbance at 240 nm. All measurements were performed in triplicate.

#### 2.8.2 DNA extraction and sequencing

Total genomic DNA was extracted from soil using the OMEGA Mag-Bind Soil DNA Kit. DNA quality was assessed using a Qubit™ 4 Fluorometer and checking integrity via 1% agarose gel electrophoresis, which showed clear high-molecular-weight band. Metagenomic libraries were prepared with the Illumina TruSeq Nano DNA LT Kit and sequenced on an Illumina NovaSeq platform (PE150) at Personal Biotechnology Co., Ltd. (Shanghai, China). The raw metagenomic sequencing data have been deposited in the NCBI Sequence Read Archive (SRA) under BioProject accession number PRJNA1417586.

#### 2.8.3 Metagenomic data analysis

Raw sequencing reads were quality-filtered using fastp v0.20.0 (Chen et al., 2018), and host DNA was removed by mapping to the *Nicotiana tabacum* reference genome using BWA-MEM (Li and Durbin, 2009). Taxonomic profiling was performed with Kraken2 (Wood et al., 2019, p. 2) against a custom RefSeq-derived database, and species-level abundances were estimated using Bracken (Lu et al., 2017). Beta-diversity was assessed by PCoA and NMDS based on Bray-Curtis distances using the vegan package in R (Dixon, 2003). Differential abundance analysis was conducted with DESeq2 (Love et al., 2014).

### 2.9 Statistical analysis

Data were analyzed using SPSS 20.0. One-way ANOVA and Duncan’s test were used for multiple comparisons; independent-sample t-tests were applied for two-group comparisons. A *p*-value < 0.05 was considered significant.

## 3 Results

### 3.1 Antifungal activity of the biocontrol strain *Streptomyces* sp. SX92^T^ against tobacco fungal pathogens

**Figure 1:**
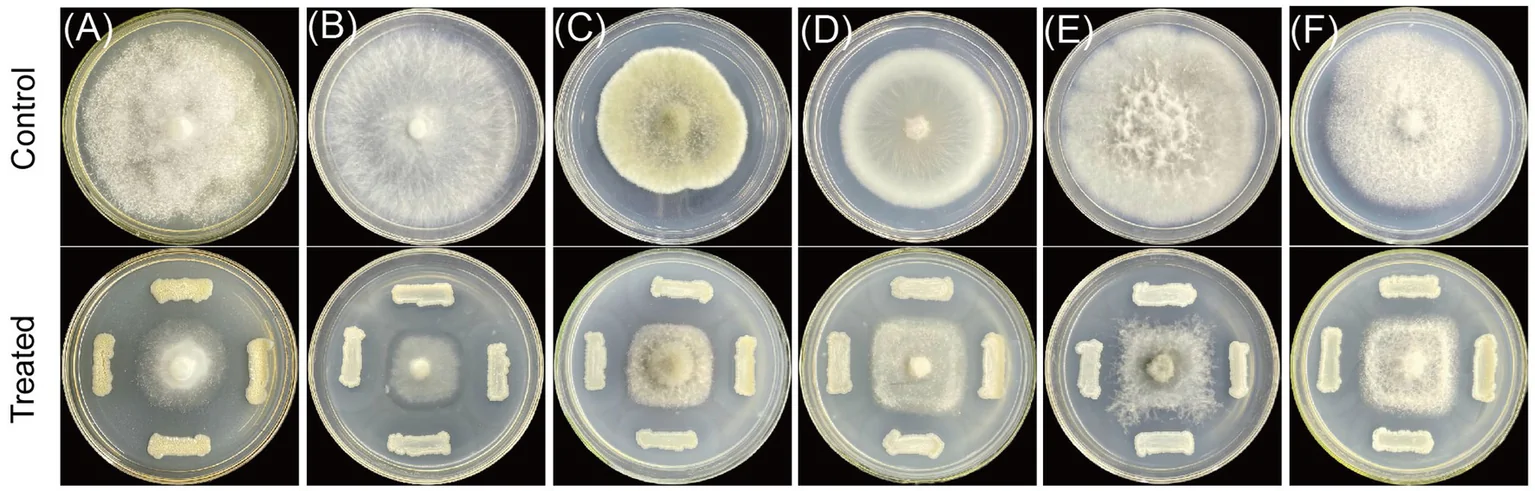
Antifungal activity of SX92^T^ against a broad spectrum of tobacco fungal pathogens. Dual culture assay of the biocontrol strain and pathogenic fungi **(A–F)** Control (top): Normal mycelia. Treated (bottom): Growth inhibition. All assays were performed on 90 mm Petri dishes. **(A)** *P. nicotianae*, **(B)** *R. solani*, **(C)** *A. alternata*, **(D)** *F. oxysporum*, **(E)** *B. dothidea*, **(F)** *F. solani*.

The antifungal activity of strain SX92^T^ against major fungal pathogens of tobacco was assessed using a dual-culture assay. SX92^T^ exhibited varying degrees of inhibition against all six pathogens tested (Figure 1). The highest inhibition rate was observed against *P. nicotianae* (59.22%), followed by *R. solani* (56.78%), *A. alternata* (48.91%), *F. oxysporum* (46.10%), *B. dothidea* (41.22%), and *F. solani* (38.34%) (Supplementary Table S1). These results demonstrate that strain SX92^T^ exhibits broad-spectrum antifungal activity.

### 3.2 Phylogenetic analysis based on 16S rRNA gene

**Figure 2:**
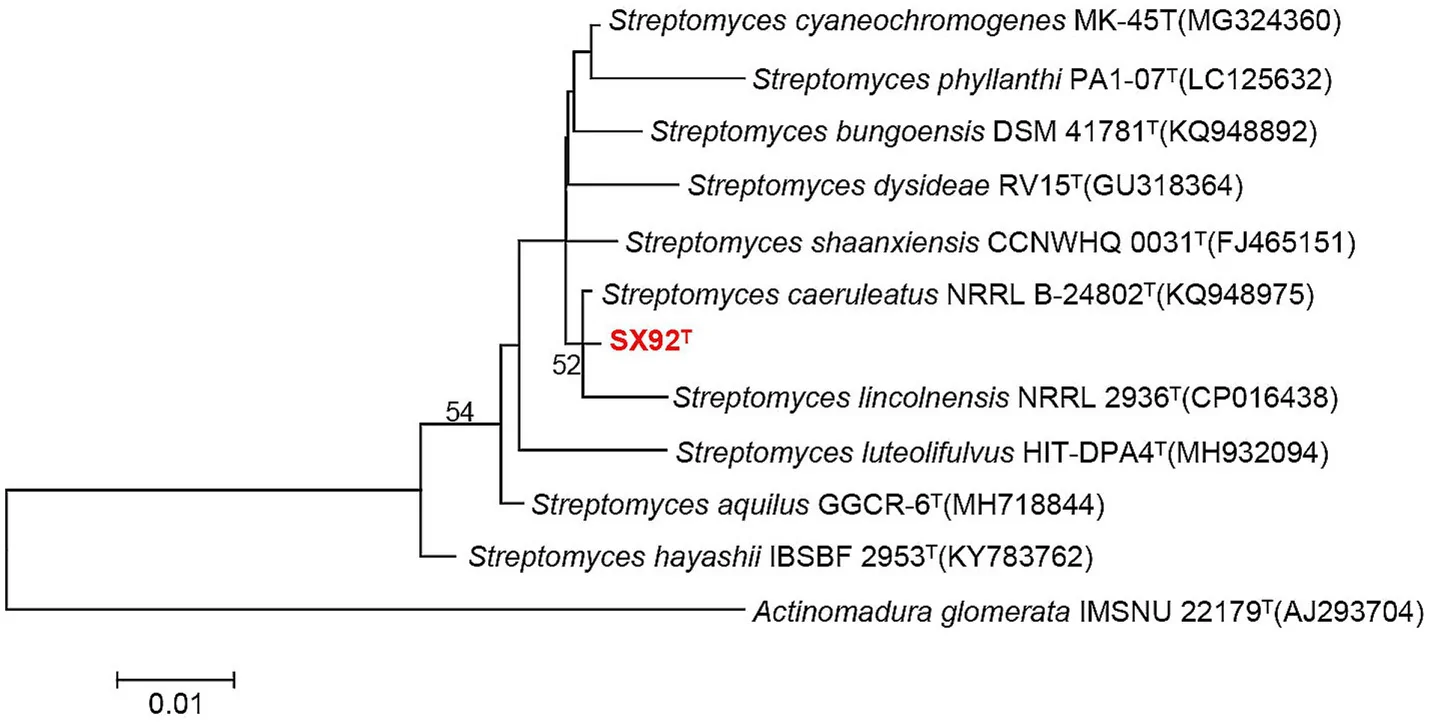
Phylogenetic tree based on 16S rRNA gene sequences. Phylogenetic tree inferred by Maximum Likelihood method. Note: The percentage of replicate trees in which the associated taxa clustered together in the bootstrap test (1,000 replicates) are shown next to the branches. The tree is drawn to scale, with branch lengths in the same units as those of the evolutionary distances used to infer the phylogenetic tree.

To determine the taxonomic status of strain SX92ᵀ, a phylogenetic tree was constructed based on its 16S rRNA gene sequence using the maximum-likelihood method. The analysis revealed that SX92ᵀ formed a distinct branch while grouping within a clade containing *Streptomyces lincolnensis* NRRL 2936ᵀ and *Streptomyces caeruleatus NRRL* B-24802ᵀ, preliminarily confirming its affiliation with the genus *Streptomyces* (Figure 2). The 16S rRNA gene sequence similarities between SX92ᵀ and these closest relatives were 98.06% (with *S. lincolnensis*) and 98.40% (with *S. caeruleus*). This topology was consistently supported in supplementary phylogenetic trees reconstructed using the maximum-parsimony and neighbour-joining methods (Supplementary Figure S1). Based on this phylogenetic proximity and the similarity values, *S. lincolnensis* and *S. caeruleatus* were selected as reference type strains for subsequent polyphasic taxonomic comparisons.

### 3.3 Chemotaxonomic study

Chemotaxonomic analyses identified rhamnose and glucose as the primary whole-cell hydrolysate sugars; LL-DAP as the major cell wall amino acid; phospholipids including diphosphatidylglycerol, phosphatidylethanolamine, phosphatidylcholine, phosphatidylinositol, phosphatidylglycerol, and unidentified phospholipids (Supplementary Figure S2); menaquinones dominated by MK-10(H₄) and MK-9(H₈) (Supplementary Figure S4; Supplementary Table S4); and fatty acids predominantly comprising anteiso-C_15:0_ and C_16:0_, (Supplementary Table S2). These characteristics are consistent with the description of the genus *Streptomyces*, further supporting its genus-level classification from a chemotaxonomic perspective.

### 3.4 Genomic features and functional potential of *Streptomyces* sp. SX92^T^

**Figure 3:**
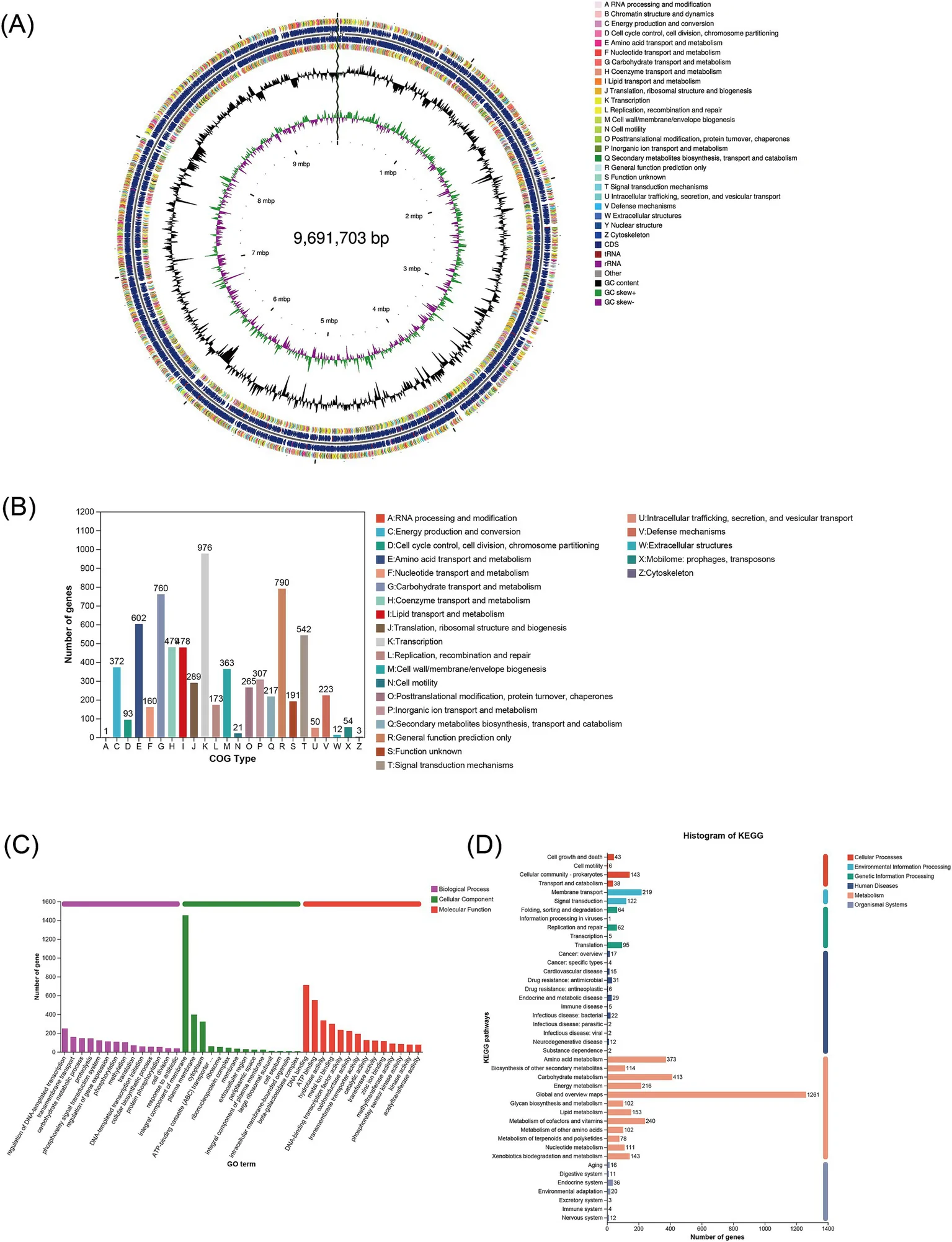
Genomic features and functional annotation of SX92^T^ **(A)** circular genome map of SX92^T^. From outer to inner: Circle 1&4: Protein-coding sequences (CDS) on the forward and reverse strands, respectively, with colors representing different COG functional categories. Circle 2&3: CDS, tRNA, and rRNA on the forward and reverse strands, respectively. Circle 5: GC content (peaks indicate deviation from average GC content). Circle 6: GC skew. Innermost circle: Genome scale (Mb). **(B)** eggNOG annotation of SX92^T^ genome. **(C)** GO annotation of SX92^T^ genome. **(D)** KEGG pathway annotation of SX92^T^.

The genomic of SX92^T^ was sequenced and functionally annotated to elucidate its characteristics and potential traits. The genome consists of a single chromosome of 9,691,730 bp with a G + C content of 71%, encoding 8,515 predicted genes, 18 rRNAs, 50 sRNAs, and 66 tRNAs (Figure 3A). Annotation using the EggNOG (COG) database classified 6,534 genes (76.74%), with significant proportions related to transcription (14.94%), carbohydrate metabolism (11.63%), and amino acid metabolism (9.21%), indicating SX92^T^ ‘s metabolic potential in these processes (Figure 3B). GO database annotation identified 5,607 genes (65.85%) associated with cellular components (e.g., intrinsic membrane proteins, cytoplasmic membrane) and molecular functions (e.g., DNA binding, ATP binding) (Figure 3C). KEGG pathway analysis further annotated 3,245 genes (38.11%) to 42 metabolic pathways, with significant enrichment in carbohydrate and amino acid metabolism, suggesting high metabolic activity in SX92^T^ (Figure 3D). Additionally, CAZy database analysis identified 178 glycoside hydrolases and other carbohydrate active enzymes (Supplementary Figure S3). AntiSMASH predicted 26 secondary metabolite biosynthetic gene clusters (BGCs). Among these, one BGC exhibited 100% similarity to the known albaflavenone biosynthetic cluster (a tricyclic sesquiterpene antibiotic)(Zheng et al., 2016) and another showed 83% similarity to the BGC for teichomycin (a glycopeptide antibiotic) (Sosio et al., 2004). Genomic analysis also identified gene clusters with high similarity to those involved in the biosynthesis of siderophores (e.g., desferrioxamine B, coelichelin)(Hesketh et al., 2007) and the osmoprotectant ectoine, suggesting potential roles in iron acquisition and stress tolerance (Nakayama et al., 2000), respectively. However, the actual production and functional contribution of these compounds require further experimental validation. (Table 1).

**Table 1:** Secondary metabolite gene clusters of SX92^T^.

Cluster ID	Type	Similar cluster	Similarity (%)
Cluster1	NRPS-like	Indigoidine	80
Cluster2	NRPS-like	Lagunapyrone A/lagunapyrone B/lagunapyrone C	22
Cluster3	Terpene	Hopene	92
Cluster4	NRPS	Coelichelin	100
Cluster5	Siderophore	–	–
Cluster6	NAPAA	Medermycin	16
Cluster7	Terpene	γ-butyrolactone	100
Cluster8	Butyrolactone	Methylenomycin A	19
Cluster9	RiPP-like	–	–
Cluster10	Phosphoglycolipid	Teichomycin	83
Cluster11	Ectoine	Kosinostatin	6
Cluster12	Siderophore	–	–
Cluster13	NRPS	BD-12	17
Cluster14	Terpene	Albaflavenone	100
Cluster15	T1PKS	Nosiheptide	30
Cluster16	NRPS	Friulimicin A/friulimicin B/Friulimicin C/friulimicin D	75
Cluster17	Ladderane	Colabomycin E	11
Cluster18	Siderophore	Desferrioxamin B/desferrioxamine E	83
Cluster19	Melanin	Istamycin	5
Cluster20	Ectoine	Ectoine	100
Cluster21	NAPAA	–	–
Cluster22	Terpene	Pradimicin-A	7
Cluster23	T3PKS	Herboxidiene	8
cluster24	Ladderane	RP-1776	12
Cluster25	NRPS	Cysteoamide	100
Cluster26	Terpene	MELANIN	57

This study uncovered the significant functional traits of the SX92^T^ genome in transcription regulation, carbohydrate and amino acid metabolism, and secondary metabolite synthesis, providing a foundation for further functional exploration and biotechnological applications.

### 3.5 Phenotypic characterization and Polyphasic taxonomic comparison

**Figure 4:**
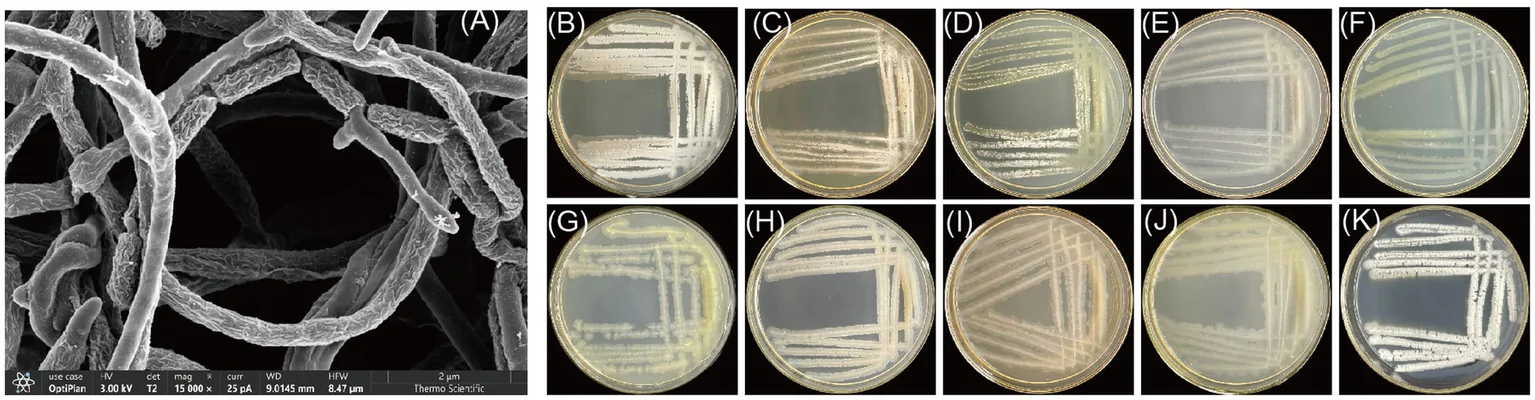
Morphological characterization of SX92^T^. **(A)** Scanning electron micrographs of hyphae and spores (35,000 × magnification). **(B–K)** Colony morphology on ISP1-ISP7, PDA, GM1, and NA.

Strain SX92ᵀ grew robustly on ISP1, ISP7, and GM1, moderately on ISP2 and ISP3, and poorly on ISP4, ISP5, ISP6, NA, and PDA. On GM1 medium, white aerial hyphae and lemon-yellow diffusible pigments were observed, whereas no soluble pigments were observed on any other media tested (Figures 4B–K). Electron microscopy observations revealed that SX92ᵀ produces well-developed, extensively branched aerial hyphae with wrinkled surfaces and non-motile rod-shaped spores arranged in wavy or flexuous chains (Figure 4A), confirming morphological traits typical of the genus *Streptomyces*.

A comparative analysis was conducted between strain SX92^T^ and the closely related type strains *S. caeruleatus* NRRL B-24802^T^ and *S. lincolnensis* NRRL 2936^T^ (Jiang et al., 2020) (Table 2). Regarding growth conditions, SX92^T^ shared a similar temperature range (5–40 °C) with *S. caeruleatus*, but differed in pH tolerance (SX92^T^: 4–10; *S. caeruleatus*: 3–9) and NaCl tolerance (SX92^T^: 0–0.3%; *S. caeruleatus*: 0.2–2.5%). In contrast, *S. lincolnensis* showed a narrower temperature range (10–37 °C) and significantly higher NaCl tolerance (0–7%). Carbon source utilization patterns distinguished SX92^T^ from both reference strains, particularly in its inability to utilize D-galactose, inositol, and lactose. Nitrogen source utilization further differentiated SX92^T^: it utilized L-arginine, unlike *S. caeruleatus*, and creatine, unlike *S. lincolnensis*. Physiologically, SX92^T^ hydrolyzed starch, while both reference strains did not. Both SX92^T^ and *S. caeruleatus* decomposed cellulose, whereas *S. lincolnensis* did not (Supplementary Figure S6). Comparative chemotaxonomy provided additional evidence for its distinctness. Although the major menaquinone composition of SX92^T^ was similar to that of *S. caeruleatus* (Supplementary Figures S4, S5; Supplementary Tables S4, S5), their polar lipid profiles differed (absence of phosphatidylinositol mannosides in SX92^T^) (Supplementary Figure S2).

**Table 2:** Comparative analysis of cultural, physiological, biochemical, and chemotaxonomic characteristics among SX92^T^ and reference strains *S. caeruleatus* NRRL B-24802^T^ and *S. lincolnensis* NRRL 2936^T.^

Characteristics	SX92^T^	*S. caeruleatus* NRRL B-24802^T^	*S. lincolnensis* NRRL 2936^T^
Growth at/with			
Temperature (°C)	5–40	5–40	10–37
pH range	4–10	3–9	5–10
Tolerance of NaCl (%, w/v)	0–3	0.2–2.5	0–7
Carbon source			
D-fructose	+	+	+
D-galactose	−	−	+
Inositol	−	+	+
Lactose	−	+	+
Maltose	+	+	+
D-mannose	−	+	+
Raffinose	+	+	+
L-rhamnose	+	+	+
D-sorbitol	+	−	+
Nitrogen source			
L-arginine	+	−	+
Creatine	+	+	−
serine	+	+	+
L-proline	+	+	+
Physiological characteristics			
Liquefaction of gelatin	+	+	−
Hydrolysis of starch	+	−	−
Peptonization in milk	−	−	−
Acid production of milk	+	+	−
Decomposition of cellulose	+	+	−
Chemotaxonomic properties			
Polar lipids profile	DPG, PE, PI, PG, PL1, PL2, GL	DPG, PE, PI, PG, PIM, PL1, PL2, PL3, GL	ND
Major fatty acids (>10%)	anteiso-C_15:0_, C_16:0_	C_15:0_, anteiso-C_15:0_, C_16:0_	anteiso-C_15:0_, C_16:0_, iso-C_16:0_, C_18:0_
Major menaquinones	MK-10(H_4_), MK-9(H_8_), MK-9(H_6_), MK-9(H_4_), MK-9(H_2_), MK-7, MK-6	MK-10(H_4_), MK-9(H_8_), MK-9(H_6_), MK-9(H_4_)	ND

−, Negativ; +, positive; “ND” no data.

For precise species delineation, the ANI and digital dDDH values between SX92ᵀ and related type strains were calculated (Table 3). The genomic comparison showed that both the ANI (83.40–92.41%) and dDDH (26.10–49.00%) values between SX92ᵀ and all reference strains were well below the accepted species boundary thresholds (ANI < 96.7%, dDDH < 70%).

**Table 3:** ANI and dDDH values between strain SX92^T^ and reference strains.

Strain	ANI	dDDH
*Streptomyces lincolnensis* NRRL 2936^T^	92.41%	49.00%
*Streptomyces caeruleatus* NRRL B-24802^T^	86.16%	27.90%
Streptomyces aquilus GGCR-6^T^	84.96%	28.50%
Streptomyces luteolifulvus HIT-DPA4^T^	84.63%	27.40%
Streptomyces hayashii IBSBF 2953^T^	83.95%	27.10%
Streptomyces shaanxiensis CCNWHQ 0031^T^	84.88%	28.00%
Streptomyces dysideae RV15^T^	85.2%	29.00%
Streptomyces cyaneochromogenes MK-45^T^	85.05%	28.00%
Streptomyces phyllanthi PA1-07^T^	83.40%	26.10%
*Streptomyces bungoensis* DSM 41781^T^	83.41%	26.30%

Based on the distinct phenotypic and chemotaxonomic characteristics, significant genomic divergence (ANI and dDDH values below species thresholds), and phylogenetic analysis, strain SX92ᵀ is concluded to represent a novel species of the genus *Streptomyces*, for which the name *Streptomyces songxianensis* sp. nov. is proposed.

### 3.6 Description of *Streptomyces songxianensis* sp. nov

*Streptomyces songxianensis* sp. nov., type strain SX92ᵀ form well-developed and extensively branched substrate and aerial mycelia. Aerial hyphae differentiate into straight to flexuous spore chains with rod-shaped, non-motile spores. Grows at 5–40 °C (optimum 25–30 °C), pH 4–10 (optimum pH 8.0), and in the presence of 0–3% (w/v) NaCl (optimum 1–1.6%). Good growth occurs on ISP1, ISP7, and GM1; moderate growth on ISP2 and ISP3; poor growth on ISP4, ISP5, ISP6, NA, and PDA. Diffusible lemon-yellow pigment is produced on GM1. Positive for starch hydrolysis, gelatin liquefaction, cellulose decomposition, and milk coagulation; negative for milk peptonization. Utilizes D-fructose, D-glucose, L-arabinose, L-rhamnose, raffinose, soluble starch, sorbitol, sucrose, and maltose as sole carbon sources. Utilizes ammonium sulfate, creatine, glycine, L-arginine, L-proline, L-methionine, L-phenylalanine, serine, and ammonium oxalate as sole nitrogen sources. Cell wall contains LL-diaminopimelic acid. Whole-cell hydrolysates contain rhamnose and glucose. The predominant menaquinones are MK-10(H₄) and MK-9(H₈). Major polar lipids are diphosphatidylglycerol, phosphatidylethanolamine, phosphatidylcholine, phosphatidylinositol, phosphatidylglycerol, and several unidentified phospholipids. The major fatty acids (>10%) are anteiso-C₁₅:₀ and C₁₆:₀.

*Streptomyces songxianensis* sp. nov., type strain SX92ᵀ (= GDMCC 4.452^T^ = JCM 37854^T^), isolated from the rhizosphere soil of a healthy tobacco plant in Luoyang, Henan Province, China. The GenBank accession numbers for the 16S rRNA gene and whole-genome sequences are MH265970.1 and CP132593, respectively.

### 3.7 Field evaluation of growth promotion in tobacco by *Streptomyces* sp. SX92^T^

**Figure 5:**
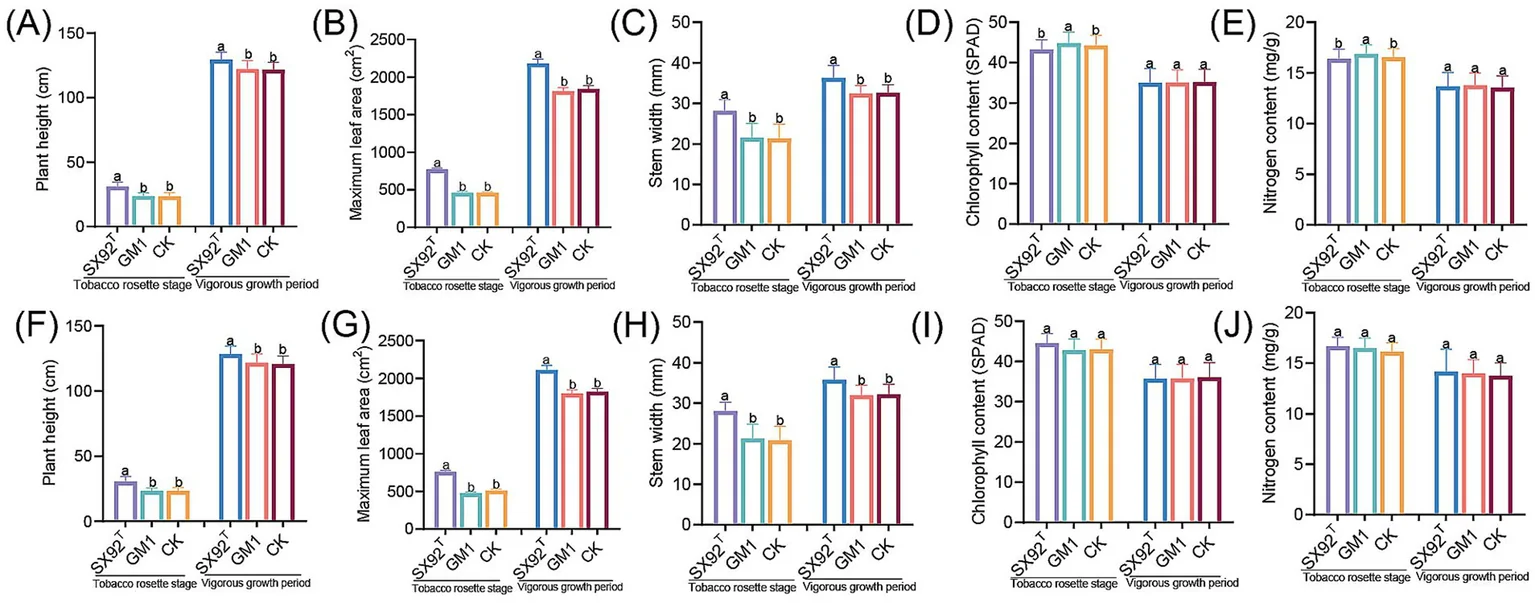
The growth-promoting effect of SX92^T^ fermentation broth on tobacco at rosette stage and vigorous growth stage. **(A–E)** Maximum leaf area, plant height, stem circumference, chlorophyll content, and nitrogen content during the rosette and vigorous growth stages in field conditions for Group A; **(F–J)** Corresponding measurements for Group B.

Field experiments conducted during the rosette and vigorous growth stages demonstrated that treatment with SX92^T^ significantly improved key agronomic traits in tobacco. During the rosette stage, SX92^T^ application markedly enhanced plant height, maximum leaf area, and stem circumference across two independent field trials (Groups A and B), though its effects on chlorophyll and nitrogen content varied between trial sites. In Group A, the maximum leaf area of the SX92^T^ treatment group increased by 67.77 and 66.82% compared to the CK control and GM1 fermentation broth control group, respectively, while plant height increased by 30.78 and 30.76%, and stem circumference increased by 31.57 and 30.76%. However, chlorophyll content and nitrogen content decreased by 3.52 and 3.02%, respectively (Figures 5A–E). In Group B, the maximum leaf area of the SX92^T^ treatment group increased by 48.36 and 60.33% compared to the CK control group and GM1 fermentation broth control group, respectively, while plant height increased by 31.68 and 30.67%, and stem circumference increased by 34.8 and 32.01%. Chlorophyll content increased by 3.58 and 4.05%, and nitrogen content increased by 3.26% (Figures 5F–J). During the vigorous growth stage, the plant height, maximum leaf area, and stem circumference of the SX92^T^ treatment group also increased significantly, but there were no significant differences in chlorophyll and nitrogen content compared to the control groups (Figure 5). Based on these results, SX92^T^ significantly promoted the growth of tobacco plants, with more pronounced effects during the rosette stage.

### 3.8 Biocontrol efficacy of *Streptomyces* sp. SX92^T^ against tobacco black shank in field trials

**Figure 6:**
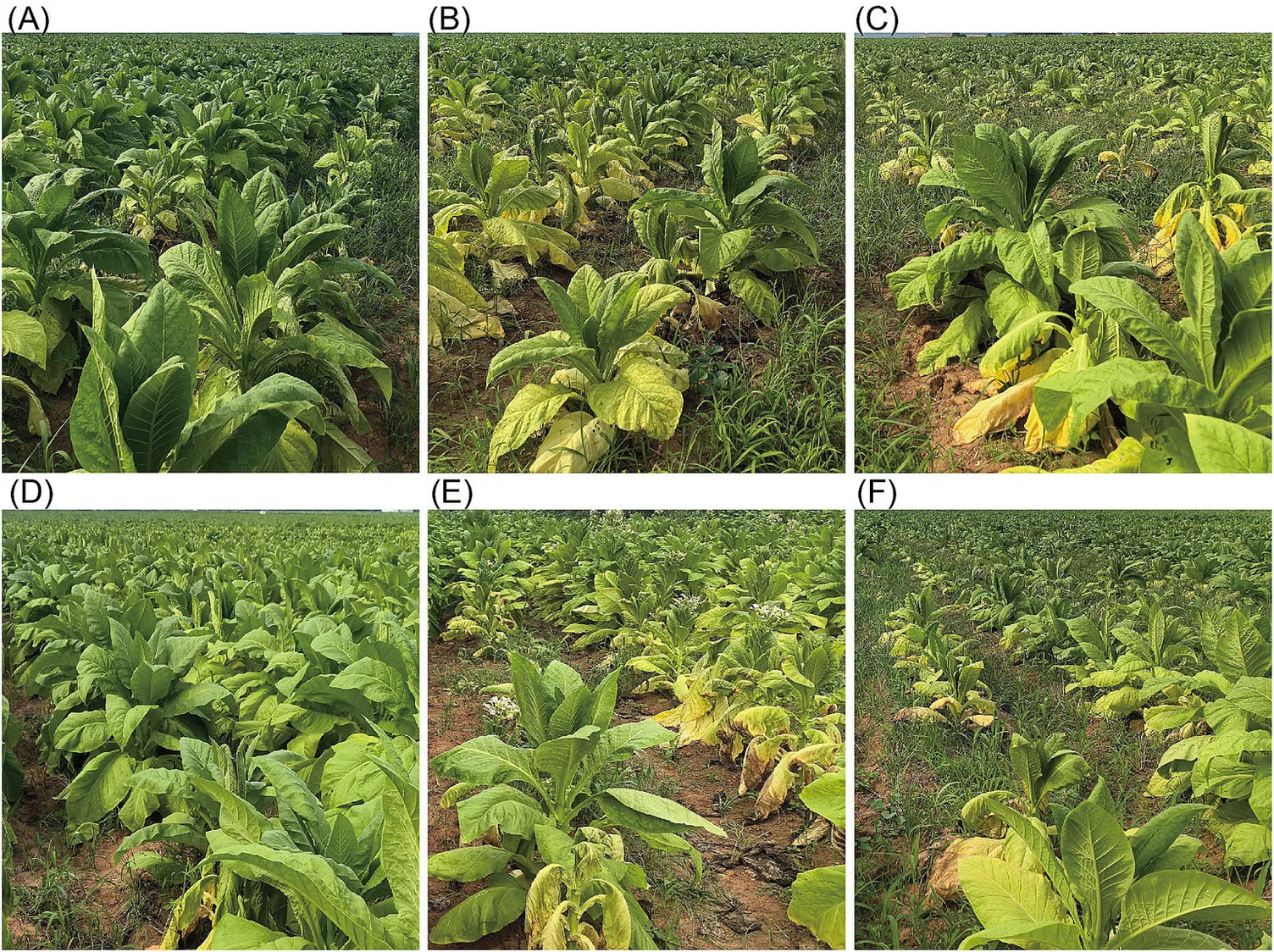
Symptoms of black shank on tobacco plants under different treatments in field experiments. Group A: **(A)** SX92^T^ treated, **(B)** GM1 fermentation broth control, **(C)** Untreated control (CK); Group B: **(D)** SX92^T^ treated, **(E)** GM1 fermentation broth control, **(F)** Untreated control (CK).

The efficacy of SX92^T^ fermentation broth in controlling tobacco black shank disease was evaluated under field conditions. At the maturity stage, severe disease incidence was observed in both control groups (CK and GM1 fermented), with typical symptoms including black, sunken lesions at the stem base and yellowing, wilting leaves adhering to the stems (Figure 6).

In Group A, treatment with SX92^T^ fermentation broth resulted in a control efficacy of 44.97% against tobacco black shank. The disease index was significantly lower than that of both the CK and GM1 fermented groups (Table 4). Similarly, in Group B, SX92^T^ treatment showed a control efficacy of 33.14%, with a significantly reduced disease index compared to the control groups (Table 4). These results demonstrate that SX92^T^ is an effective biocontrol strain against tobacco black shank disease.

**Table 4:** Control effects of SX92^T^ fermentation broth on tobacco black shank in field.

Group	Treatment	Field control efficiency		
		Incidence rate/%	Disease index	Control effect/%
GroupA	SX92^T^	28.67 ± 1.33b	12.856 ± 1.31b	44.97 ± 2.8
	GM1	44 ± 4a	20.914 ± 1.91a	–
	CK	49.33 ± 2.45a	23.202 ± 1.55a	–
GroupB	SX92^T^	29.33 ± 0.67b	13.92 ± 1.99b	33.14 ± 5.47
	GM1	47.33 ± 1.63a	20.21 ± 1.22a	–
	CK	43.33 ± 2.79a	20.7 ± 1.94a	–

Values within a column followed by different lowercase letters differ significantly at *p* < 0.05 (Duncan’s multiple range test). The “–” symbol indicates exclusion of positive blank control from statistical analysis.

### 3.9 Effects of *Streptomyces* sp. SX92^T^ on rhizosphere soil enzyme activities

**Figure 7:**
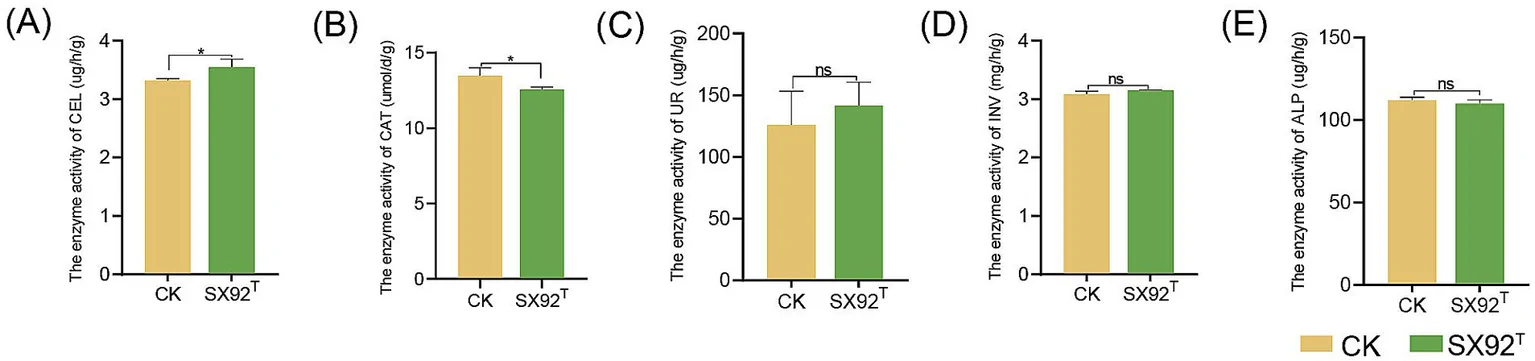
Soil enzyme activities under CK and SX92^T^ treatments. **(A)** Cellulase activity in rhizosphere soil; **(B)** Catalase activity in rhizosphere soil; **(C)** Urease activity; **(D)** Sucrase activity; **(E)** Alkaline phosphatase activity. Different lowercase letters indicate significant differences between treatments at *p* < 0.05 (Duncan’s test). *ns* = not significant.

This study investigated the effects of SX92^T^ treatment on soil enzyme activities in comparison with the control group (CK). The results showed no significant differences (*p* > 0.05) in urease, sucrase, or alkaline phosphatase activities between the two groups (Figures 7C–E). However, the SX92^T^ treatment significantly enhanced cellulase activity (*p* < 0.05) while reducing catalase activity (*p* < 0.05) in the rhizosphere soil relative to CK (Figures 7A,B). These findings indicate that SX92^T^ treatment altered specific soil enzyme activities, suggesting a potential shift in microbial functional processes related to organic matter decomposition and redox dynamics. The increased cellulase activity suggests enhanced decomposition of organic matter and improved nutrient cycling, while the decreased catalase activity may reflect reduced oxidative stress in the modulated microbial community (Burns et al., 2013; Trasar-Cepeda et al., 1999).

### 3.10 High-throughput sequencing analysis of rhizosphere microbiota following *Streptomyces* sp. SX92^T^ treatment

High-throughput sequencing of rhizosphere soil samples from SX92^T^ treated and control groups produced high quality data, with sequencing depths reaching 12 Gb per sample. The three biological replicates of the control group (CK-1 to CK-3) displayed contig counts ranging from 519,914 to 607,709, total base pairs from 355,430,763 to 395,259,201 bp, and N50 values between 621 and 670 bp. In contrast, the SX92^T^ treated sample SX92^T^-3, while exhibiting the lowest contig count (174,347), demonstrated superior assembly quality metrics, including the longest contig (944,060 bp) and highest N50 value (4,050 bp) among all samples (Supplementary Table S6).

### 3.11 SX92^T^ treatment restructures soil microbial communities by altering bacterial dominance and suppressing pathogenic Fungi

**Figure 8:**
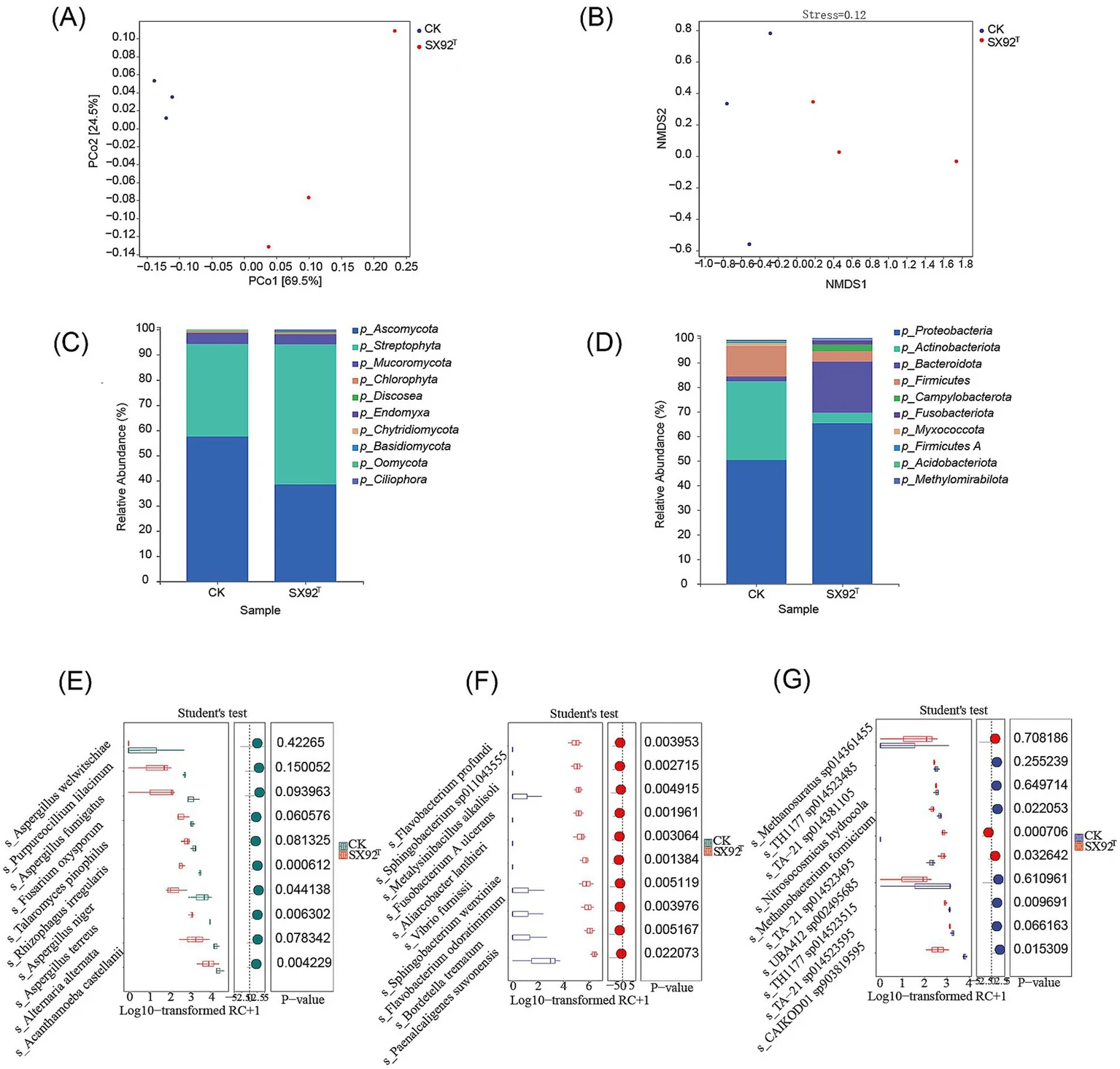
Effects of SX92^T^ on the structure and composition of tobacco rhizosphere soil microbial communities. **(A)** Principal coordinates analysis (PCoA) plot based on Bray-Curtis distances; **(B)** Non-metric multidimensional scaling (NMDS) plot based on Bray-Curtis distances; **(C)** Composition of the top 10 phyla in fungal relative abundance; **(D)** Composition of the top 10 phyla in bacterial relative abundance; **(E)** Differential significance of the top 10 fungal species in relative abundance; **(F)** Differential significance of the top 10 bacterial species in relative abundance; **(G)** Differential significance of the top 10 archaeal species in relative abundance.

To evaluate the comprehensive impact of SX92^T^ treatment on the soil microbial community, this study focused on inter-community structural differences (*β*-diversity). β-diversity analysis revealed treatment-induced shifts in the overall community structure. Principal coordinate analysis (PCoA) based on Bray–Curtis distances indicated significant differences in microbial community structure among the treatments (Figure 8A). As shown in the plot, samples from the control group and the SX92^T^ -treated group were distinctly separated and distributed in different quadrants, suggesting that the introduction of *Streptomyces* sp. SX92^T^ significantly altered the composition of the soil microbial community. Non-metric multidimensional scaling (NMDS) yielded consistent results (Figure 8B), with a stress value of 0.12, indicating that the two-dimensional ordination plot reliably reflected the actual differences among samples.

Regarding the effect of the SX92^T^ inoculant on soil microbial community structure, analysis of the top 10 abundant fungal and bacterial phyla showed that at the fungal phylum level, the relative abundance of Streptophyta was significantly increased in the treatment group, while that of Ascomycota decreased (Figure 8C). At the bacterial phylum level, the relative abundances of Proteobacteria and Bacteroidota were significantly elevated in the treated soil, while those of Firmicutes and Actinobacteriota showed a relative decrease (Figure 8D). At the species level, analysis of differential abundance revealed a significant reduction in the relative abundance of certain fungal taxa, including the arbuscular mycorrhizal fungus *Rhizophagus irregularis* (*p* = 0.000612), and the saprophytes *Aspergillus niger* (*p* = 0.044138) and *A. terreus* (*p* = 0.006302) (Figure 8E); bacterial assessment revealed nine taxa exhibiting extremely significant enrichment in SX92^T^ (all *p* < 0.01), including *Flavobacterium profundi* (*p* = 0.003953), *Sphing obacterium* sp011043555 (*p* = 0.002715), and *Metalysinibacillus alkalisoli* (*p* = 0.004915) (Figure 8F); archaeal communities maintained structural stability with no significant alterations (*p* > 0.05; Figure 8G). These results indicate that SX92^T^ treatment was associated with significant alterations in the soil microbial structure. It should be noted that these changes may result from a combination of direct antagonism by SX92^T^, plant-mediated recruitment of beneficial taxa due to improved plant health, and altered root exudation pattern.

## 4 Discussion

This study establishes *Streptomyces songxianensis* SX92^T^ as a novel species exhibiting antagonistic activity against major fungal pathogens of tobacco, most notably against the economically significant pathogen *P. nicotianae*. Genomic analysis revealed substantial potential for secondary metabolite biosynthesis. Among the predicted BGC, one exhibited 100% similarity to the albaflavenone cluster. Furthermore, gene clusters with high similarity to those involved in siderophore (e.g., desferrioxamine B, coelichelin) and osmoprotectant ectoine biosynthesis were identified, suggesting potential roles in iron acquisition and stress tolerance, respectively. These genetic features, combined with its demonstrated efficacy in disease suppression and plant growth promotion in field trials, provide a foundational basis for understanding the strain’s multifunctional potential. However, the precise chemical identity and functional contribution of these predicted metabolites *in situ* require further biochemical and genetic validation. Field trials confirmed the strain’s dual functionality, demonstrating significant enhancement of tobacco plant growth through traits such as a 67.8% increase in leaf area, along with effective control of black shank disease showing 44.97% efficacy. Moreover, application of SX92^T^ fermentation broth induced beneficial restructuring of the rhizosphere microbiome, such as enrichment of *Proteobacteria* and suppression of pathogenic communities - along with modulation of soil enzyme activities, notably increased cellulase and decreased catalase, indicating its role in promoting a healthier root microenvironment.

The antagonistic activity of SX92^T^ against *P. nicotianae* and other pathogens is likely attributable to its capacity to produce diverse antimicrobial metabolites. The identification of 26 BGCs in its genome provides a genetic basis for this metabolic potential. Notably, although the anti-oomycete metabolite borrelidin is known to be synthesized by some streptomycetes, (Liu et al., 2012; Zhou et al., 2024), no homologous BGC was identified in SX92^T^, This suggests that the anti-oomycete activity of SX92^T^ may be mediated by one or more previously unrecognized compounds—a finding that opens promising avenues for natural product exploration. The observed dual effects of growth promotion (at the rosette stage) and disease suppression under field conditions align with established mechanisms by which streptomycetes enhance plant growth, including phytohormone modulation, nutrient mobilization, and induced resistance (Patel et al., 2018). It is important to note, however, that the efficacy of streptomycete biocontrol agents in the field can be variable and influenced by environmental and edaphic factors, which may affect the expression of specific BGCs and the production of corresponding metabolites (Kaari et al., 2023).

Treatment with SX92^T^ also markedly altered the structure of the rhizosphere microbial community and soil enzyme activities. Enrichment of *Proteobacteria*, a phylum encompassing multiple plant-beneficial genera such as *Pseudomonas* and *Bacillus*, coupled with suppression of potentially pathogenic fungi including *A. niger* and *A. terreus*, suggests that SX92^T^ may facilitate the recruitment of beneficial microbial alliances that enhance plant health, consistent with the “cry for help” hypothesis (Arnault et al., 2023; Gao et al., 2021). These changes were accompanied by shifts in soil enzyme profiles: elevated cellulase activity indicating improved nutrient cycling, and reduced catalase activity suggesting modulation of rhizosphere oxidative status, further underscoring the microecological regulatory role of SX92^T^. It should be noted that the causal links between the specific enriched taxa observed in this study and the improved tobacco growth performance and disease resistance remain to be directly validated through experimental approaches such as synthetic microbial community (SynCom) experiments.

The identification of *Streptomyces songxianensis* SX92^T^ enhances the diversity of biocontrol streptomycetes and provides a solid scientific foundation for its field application. Our findings demonstrate that a single *Streptomyces* strain can deliver multi-faceted benefits including pathogen inhibition, plant growth promotion, and functional restructuring of the rhizosphere microbiome under real-world agricultural conditions, advancing our mechanistic understanding of how biocontrol agents contribute to soil suppressiveness. From a practical standpoint, SX92^T^ shows considerable promise as a core component of integrated management strategies against tobacco black shank disease. Its combined disease control and growth-promoting abilities offer a sustainable alternative that could help reduce dependence on chemical pesticides and support sustainable agriculture. Future work should prioritize the development of stable, commercially viable formulations based on SX92^T^ fermentation broth to facilitate its broad application.

## 5 Conclusion

This study establishes *Streptomyces songxianensis* SX92^T^ as a novel biocontrol agent with dual functionality in pathogen suppression and plant growth promotion. *In vitro* assays demonstrated broad-spectrum antifungal activity against major tobacco pathogenic fungi, most notably a 59.22% inhibition rate against *P. nicotianae*. Polyphasic taxonomic characterization confirmed its novelty, supported by distinct phenotypic, chemotaxonomic, and genomic features, including a 9.69 Mbp genome with 26 biosynthetic gene clusters. Field trials validated its practical efficacy, revealing significant enhancements in tobacco growth, such as a 67.8% increase in leaf area, along with a 44.97% reduction in black shank disease incidence. Rhizosphere modulation analysis indicated that SX92^T^ enrichment reshapes microbial communities by promoting beneficial *Proteobacteria*, suppressing pathogens, and altering soil enzyme activities, exemplified by increased cellulase and decreased catalase levels, thereby improving soil health. These findings underscore the strain’s potential as a sustainable alternative to chemical pesticides, offering a multifaceted approach to enhancing crop resilience and soil ecological balance. Future studies should focus on industrial-scale production and the mechanistic elucidations of its bioactive compounds.

## Data Availability

The datasets presented in this study can be found in online repositories. The names of the repository/repositories and accession number(s) can be found in the article/Supplementary material.

## Footnotes

1
https://www.ezbiocloud.net/

## Supplementary material

Supplementary Figure S1 Phylogenetic tree based on 16S rRNA gene sequences. **(A)** Phylogenetic tree constructed by Neighbor-Joining method. **(B)** Phylogenetic tree inferred by Maximum parsimony method. The percentage of replicate trees in which the associated taxa clustered together in the bootstrap test (1000 replicates) are shown next to the branches. The tree is drawn to scale, with branch lengths in the same units as those of the evolutionary distances used to infer the phylogenetic tree.

Supplementary Figure S2 Biochemical characteristics of SX92T and B-24802T. **(A)** Cell wall amino acid composition of SX92^T^. **(B)** Cell wall sugar composition of SX92T. **(C)** Polar lipid profile of SX92^T^ stained with molybdatophosphoric acid. **(D)** Polar lipid profile of B-24802^T^ stained with molybdatophosphoric acid. **(E)** Polar lipid profile of SX92^T^ stained with ninhydrin. **(F)** Polar lipid profile of B-24802T stained with ninhydrin. **(G)** Polar lipid profile of SX92T stained with molybdenum blue. **(H)** Polar lipid profile of B-24802T stained with molybdenum blue. **(I)** Polar lipid profile of SX92T stained with 1-methylnaphthol. **(J)** Polar lipid profile of B-24802T stained with 1-methylnaphthol. **(K)** Polar lipid profile of SX92T stained with reagent Dragendorff. **(L)** Polar lipid profile of B-24802T stained with reagent Dragendorff.

Supplementary Figure S3 Classification of carbohydrate-active enzymes in the SX92^T^ genome. AA, Auxiliary Activities; CBM, Carbohydrate-Binding Modules; CE, Carbohydrate Esterases; GH, Glycoside Hydrolases; GT, Glycosyl Transferases; PL, Polysaccharide Lyases.

Supplementary Figure S4 Composition and content of respiratory quinones in the cellular membrane of SX92^T^.

Supplementary Figure S5 Composition and content of respiratory quinones in the cellular membrane of B-24802^T^.

Supplementary Figure S6 Growth and physiological characteristics of SX92T and B-24802T. **(A)** Growth of SX92^T^ and B-24802T at temperatures from 0 to 50 °C. **(B)** Growth of SX92^T^ and B-24802T at pH 2–12. **(C)** Growth of SX92T and B-24802T in the presence of 0–3% (w/v) NaCl. **(D)** Cellulase activity of SX92T and B-24802T. **(E)** Starch hydrolysis by SX92T and B-24802T. **(F)** Hydrogen sulfide production by SX92T and B-24802T. **(G)** Peptonization and acid production in milk by SX92T and B-24802T. **(H)** Gelatin liquefaction by SX92T and B-24802T. **(I)** Methyl red (MR) test of SX92T and B-24802T. **(J)** Voges Proskauer (VP) test of SX92T and B-24802T. **(K)** Utilization of carbon sources by SX92T and B-24802T. **(L)** Utilization of nitrogen sources by SX92T and B-24802^T^.
