# Supplementary material for: Streptomyces songxianensis sp. nov. SX92T: biocontrol of tobacco black shank and modulation of the rhizosphere microbiome
Source: Front Microbiol. 2026 Aug 25;17:1820235. doi: 10.3389/fmicb.2026.1820235 (PMC13550158; doi:10.3389/fmicb.2026.1820235)
Supplement: Supplementary file 1 [file Supplementary_file_1.docx]

**Table S1.** Inhibitory effect of SX92^T^ on the mycelial growth of tobacco fungal pathogens

| Treatment | Mycelium diameter (cm±SE) |  | Inhibition Rates (%) |
| --- | --- | --- | --- |
|  | Control Group | Treatment Group |  |
| *Phytophthora nicotianae* | 9±0.00a | 3.67±0.07b | 59.22 |
| *Rhizoctonia solani* | 9±0.00a | 3.89±0.09b | 56.78 |
| *Alternaria alternata* | 7.77±0.08a | 3.97±0.08b | 48.91 |
| *Fusarium oxysporum* | 8.07±0.05a | 4.35±0.04b | 46.10 |
| *Botryosphaeria dothidea* | 9±0.00a | 5.29±0.10b | 41.22 |
| *Fusarium solani* | 8.58±0.05a | 5.29±0.06b | 38.34 |

Note. Values within a column followed by different lowercase letters differ significantly at *P* < 0.05 (Duncan's multiple range test).
