# Supplementary material for: Streptomyces songxianensis sp. nov. SX92T: biocontrol of tobacco black shank and modulation of the rhizosphere microbiome
Source: Front Microbiol. 2026 Aug 25;17:1820235. doi: 10.3389/fmicb.2026.1820235 (PMC13550158; doi:10.3389/fmicb.2026.1820235)
Supplement: Supplementary file 2 [file Supplementary_file_2.docx]

**Table S2.** Cellular fatty acid composition of SX92^T^

| RT | Response | Ar/Ht | RFact | ECL | Peak Name | Percent | Comment1 | Comment2 |
| --- | --- | --- | --- | --- | --- | --- | --- | --- |
| 0.7072 | 1.079E+9 | 0.022 | ---- | 6.7063 | SOLVENT PEAK | ---- | < min rt |  |
| 1.6251 | 1335 | 0.013 | 1.107 | 12.0001 | C_12:0_ | 0.16 | ECL deviates 0.000 | Reference 0.000 |
| 1.7916 | 2031 | 0.013 | 1.076 | 12.6242 | iso-C_13:0_ | 0.24 | ECL deviates 0.001 | Reference 0.002 |
| 1.8156 | 2371 | 0.013 | 1.072 | 12.7141 | anteiso-C_13:0_ | 0.28 | ECL deviates 0.000 | Reference 0.001 |
| 1.8916 | 1080 | 0.014 | 1.058 | 12.9991 | C_13:0_ | 0.13 | ECL deviates -0.001 | Reference 0.000 |
| 2.0756 | 20144 | 0.012 | 1.030 | 13.6280 | iso-C_14:0_ | 2.40 | ECL deviates 0.000 | Reference 0.000 |
| 2.1840 | 16758 | 0.012 | 1.014 | 13.9986 | C_14:0_ | 2.00 | ECL deviates -0.001 | Reference -0.001 |
| 2.3791 | 47258 | 0.012 | 0.988 | 14.6310 | iso-C_15:0_ | 5.64 | ECL deviates -0.001 | Reference -0.001 |
| 2.4081 | 183140 | 0.013 | 0.985 | 14.7252 | anteiso-C_15:0_ | 21.85 | ECL deviates 0.000 | Reference 0.000 |
| 2.4539 | 3096 | 0.012 | 0.979 | 14.8736 | C_15:1_ω6*c* | 0.37 | ECL deviates -0.001 |  |
| 2.4930 | 29744 | 0.012 | ---- | 15.0005 | C_15:0_ | 3.55 | ECL deviates 0.000 |  |
| 2.6455 | 6145 | 0.013 | 0.957 | 15.4825 | iso-_16:1_ | 0.73 | ECL deviates 0.001 |  |
| 2.6934 | 63354 | 0.012 | 0.952 | 15.6339 | iso-16:0 | 7.56 | ECL deviates 0.001 | Reference 0.000 |
| 2.7583 | 112453 | 0.013 | 0.945 | 15.8391 | Summed Feature 3 | 13.42 | ECL deviates -0.001 | C₁₆:₁ ω6c/C₁₆:₁ ω7c |
| 2.8098 | 178607 | 0.012 | 0.939 | 16.0021 | C_16:0_ | 21.31 | ECL deviates 0.002 | Reference 0.001 |
| 2.8907 | 544 | 0.012 | 0.931 | 16.2575 | C_15:0_ 2-OH | 0.06 | ECL deviates 0.002 |  |
| 2.9493 | 15130 | 0.012 | 0.925 | 16.4423 | Summed Feature 9 | 1.80 | ECL deviates -0.005 | iso-C₁₇:₁ ω9c |
| 2.9852 | 22949 | 0.013 | 0.922 | 16.5557 | anteiso-C_17:1_ω9*c* | 2.74 | ECL deviates 0.004 |  |
| 3.0106 | 14481 | 0.012 | 0.920 | 16.6358 | Iso-C_17:0_ | 1.73 | ECL deviates -0.001 | Reference -0.003 |
| 3.0411 | 76076 | 0.013 | 0.917 | 16.7322 | anteiso-C_17:0_ | 9.08 | ECL deviates -0.001 | Reference -0.003 |
| 3.0675 | 10091 | 0.012 | 0.915 | 16.8154 | C₁₇:₁ ω8*c* | 1.20 | ECL deviates 0.000 |  |
| 3.0987 | 7270 | 0.014 | 0.912 | 16.9140 | C_17:0_ cyclo | 0.87 | ECL deviates -0.001 |  |
| 3.1263 | 9913 | 0.012 | 0.909 | 17.0012 | C_17:0_ | 1.18 | ECL deviates 0.001 | Reference -0.001 |
| 3.2620 | 962 | 0.014 | ---- | 17.4332 |  | 0.11 |  |  |
| 3.2803 | 724 | 0.012 | 0.896 | 17.4914 | iso-C_18:1_ | 0.09 | ECL deviates 0.001 |  |
| 3.2981 | 887 | 0.014 | 0.895 | 17.5479 | C_16:0_ 3-OH | 0.11 | ECL deviates 0.000 |  |
| 3.3258 | 812 | 0.014 | 0.893 | 17.6364 | iso-C_18:0_ | 0.10 | ECL deviates 0.000 | Reference -0.003 |
| 3.3744 | 4411 | 0.021 | 0.889 | 17.7909 | C₁₈:₁ ω9*c* | 0.53 | ECL deviates -0.003 |  |
| 3.3908 | 2772 | 0.015 | 0.888 | 17.8431 | Summed Feature 8 | 0.33 | ECL deviates -0.004 | C₁₈:₁ ω7c |
| 3.4398 | 2571 | 0.013 | 0.884 | 17.9992 | C_18:0_ | 0.31 | ECL deviates -0.001 | Reference -0.005 |
| 3.7944 | 1154 | 0.013 | 0.861 | 19.1573 | C_18:1_ 2-OH | 0.14 | ECL deviates 0.011 |  |
| ---- | 112453 | --- | ---- | ---- | Summed Feature 3 | 13.42 | 16:1 w7c/16:1 w6c | 16:1 w6c/16:1 w7c |
| ---- | 2772 | --- | ---- | ---- | Summed Feature 8 | 0.33 | C₁₈:₁ ω7c | C₁₈:₁ ω6c |
| ---- | 15130 | --- | ---- | ---- | Summed Feature 9 | 1.80 | C_16:0_ 10-methyl | iso-C₁₇:₁ ω9c |
