# Supplementary material for: Streptomyces songxianensis sp. nov. SX92T: biocontrol of tobacco black shank and modulation of the rhizosphere microbiome
Source: Front Microbiol. 2026 Aug 25;17:1820235. doi: 10.3389/fmicb.2026.1820235 (PMC13550158; doi:10.3389/fmicb.2026.1820235)
Supplement: Supplementary file 3 [file Supplementary_file_3.docx]

**Table S3.** Cellular fatty acid composition of B-24802^T^

| RT | Response | Ar/Ht | RFact | ECL | Peak Name | Percent | Comment1 | Comment2 |
| --- | --- | --- | --- | --- | --- | --- | --- | --- |
| 0.7072 | 1.074E+9 | 0.022 | ---- | 6.7075 | SOLVENT PEAK | ---- | < min rt |  |
| 1.0233 | 1029 | 0.022 | ---- | 8.8146 |  | 0.10 | < min rt |  |
| 1.2013 | 1546 | 0.017 | 1.219 | 9.9977 | C_10:0_ | 0.15 | ECL deviates -0.002 | Reference -0.002 |
| 1.3201 | 436 | 0.012 | 1.182 | 10.6185 | iso-C_11:0_ | 0.04 | ECL deviates 0.000 | Reference 0.001 |
| 1.3373 | 649 | 0.014 | 1.177 | 10.7083 | anteiso-C_11:0_ | 0.06 | ECL deviates 0.003 | Reference 0.004 |
| 1.6252 | 1162 | 0.014 | 1.107 | 11.9997 | C_12:0_ | 0.11 | ECL deviates 0.000 | Reference 0.001 |
| 1.7915 | 7502 | 0.013 | 1.076 | 12.6231 | iso-C_13:0_ | 0.72 | ECL deviates 0.000 | Reference 0.001 |
| 1.8158 | 3352 | 0.012 | 1.072 | 12.7145 | anteiso-C_13:0_ | 0.32 | ECL deviates 0.000 | Reference 0.001 |
| 1.8917 | 469 | 0.013 | 1.058 | 12.9989 | C_13:0_ | 0.04 | ECL deviates -0.001 | Reference 0.000 |
| 2.0754 | 20436 | 0.013 | 1.030 | 13.6273 | iso-C_14:0_ | 1.96 | ECL deviates -0.001 | Reference 0.000 |
| 2.1838 | 11727 | 0.012 | 1.014 | 13.9979 | C_14:0_ | 1.12 | ECL deviates -0.002 | Reference -0.002 |
| 2.3394 | 806 | 0.014 | 0.993 | 14.5026 | Summed Feature 1 | 0.08 | ECL deviates 0.000 | C_13:0_3-OH/iso-C_15:1_ |
| 2.3794 | 277066 | 0.012 | 0.988 | 14.6324 | iso-C_15:0_ | 26.54 | ECL deviates 0.000 | Reference 0.000 |
| 2.4081 | 235483 | 0.013 | 0.985 | 14.7255 | anteiso-C_15:0_ | 22.56 | ECL deviates 0.000 | Reference 0.000 |
| 2.4535 | 582 | 0.012 | 0.979 | 14.8728 | C₁₅:₁ ω6c | 0.06 | ECL deviates -0.002 |  |
| 2.4927 | 12574 | 0.012 | ---- | 14.9998 | C_15:0_ | 1.20 | ECL deviates 0.000 |  |
| 2.6452 | 9257 | 0.013 | 0.957 | 15.4820 | iso-C_16:1_ | 0.89 | ECL deviates 0.000 |  |
| 2.6934 | 77628 | 0.012 | 0.952 | 15.6343 | iso-C_16:0_ | 7.44 | ECL deviates 0.001 | Reference 0.000 |
| 2.7576 | 40389 | 0.012 | 0.945 | 15.8375 | Summed Feature 3 | 3.87 | ECL deviates -0.002 | C_16:1_ω7c/C_16:1_ω6c |
| 2.8092 | 115491 | 0.012 | 0.939 | 16.0006 | C_16:0_ | 11.06 | ECL deviates 0.001 | Reference -0.001 |
| 2.9493 | 51915 | 0.012 | 0.925 | 16.4429 | Summed Feature 9 | 4.97 | ECL deviates -0.004 | iso-C_17:1_ω9c |
| 2.9849 | 20839 | 0.014 | 0.922 | 16.5551 | anteiso-C₁₇:₁ ω9c | 2.00 | ECL deviates 0.003 |  |
| 3.0107 | 64976 | 0.012 | 0.920 | 16.6366 | iso-C_17:0_ | 6.22 | ECL deviates 0.000 | Reference -0.003 |
| 3.0412 | 62642 | 0.013 | 0.917 | 16.7330 | anteiso-C_17:0_ | 6.00 | ECL deviates 0.000 | Reference -0.003 |
| 3.0674 | 1778 | 0.012 | 0.915 | 16.8157 | C₁₇:₁ ω8c | 0.17 | ECL deviates 0.001 |  |
| 3.0980 | 1713 | 0.015 | 0.912 | 16.9125 | C_17:0_ cyclo | 0.16 | ECL deviates -0.003 |  |
| 3.1259 | 3493 | 0.012 | 0.909 | 17.0005 | C_17:0_ | 0.33 | ECL deviates 0.001 | Reference -0.002 |
| 3.2800 | 1025 | 0.017 | 0.896 | 17.4910 | iso-C_18:1_ | 0.10 | ECL deviates 0.001 |  |
| 3.3630 | 12215 | 0.013 | 0.890 | 17.7554 | Summed Feature 5 | 1.17 | ECL deviates -0.001 | C_18:2_ω6,9c/ante-C_18:0_ |
| 3.3899 | 4049 | 0.014 | 0.888 | 17.8409 | Summed Feature 8 | 0.39 | ECL deviates -0.007 | C_18:1_ω7c |
| 3.4396 | 1573 | 0.014 | 0.884 | 17.9993 | C_18:0_ | 0.15 | ECL deviates -0.001 | Reference -0.005 |
| ---- | 806 | --- | ---- | ---- | Summed Feature 1 | 0.08 | iso-C_15:1_ /C_13:0_3-OH | C_13:0_-3OH/iso-C_15:1_ |
| ---- | 40389 | --- | ---- | ---- | Summed Feature 3 | 3.87 | C₁₆:₁ ω6c/C₁₆:₁ ω7c | C₁₆:₁ω6c/C₁₆:₁ ω7c |
| ---- | 12215 | --- | ---- | ---- | Summed Feature 5 | 1.17 | anteiso-C_18:0_/C_18:2_ ω6,9c | C₁₈:₂ω6,9c/anteiso-C₁₈:₀ |
| ---- | 4049 | --- | ---- | ---- | Summed Feature 8 | 0.39 | C₁₈:₁ ω7c | C₁₈:₁ ω6c |
| ---- | 51915 | --- | ---- | ---- | Summed Feature 9 | 4.97 | C_16:0_10-methyl | iso-C₁₇:₁ ω9c |
