# Supplementary material for: Streptomyces songxianensis sp. nov. SX92T: biocontrol of tobacco black shank and modulation of the rhizosphere microbiome
Source: Front Microbiol. 2026 Aug 25;17:1820235. doi: 10.3389/fmicb.2026.1820235 (PMC13550158; doi:10.3389/fmicb.2026.1820235)
Supplement: Supplementary file 4 [file Supplementary_file_4.docx]

**Table S4.** Composition and content of respiratory quinones in the cellular membrane of SX92^T^

| Peak Number | Retention Time (min) | Peak Area (mAU·min) | Peak Height (mAU) | Relative Area (%) | Quinone Type |
| --- | --- | --- | --- | --- | --- |
| 1 | 13.493 | 81685 | 3531 | 2.212 | MK-6 |
| 2 | 14.988 | 62348 | 2446 | 1.689 | MK-7 |
| 3 | 27.7 | 72595 | 1834 | 1.966 | MK-9(H_2_) |
| 4 | 29.971 | 53051 | 1258 | 1.437 | MK-9(H_4_) |
| 5 | 31.305 | 37153 | 852 | 1.006 | MK-9(H_4_) |
| 6 | 32.86 | 233034 | 4354 | 6.312 | MK-9(H_4_) |
| 7 | 34.307 | 56883 | 1075 | 1.541 | MK-9(H_6_) |
| 8 | 37.715 | 1782404 | 30447 | 48.275 | MK-9(H_8_) |
| 9 | 42.869 | 1313014 | 20328 | 35.562 | MK10(H_4_) |
