# Supplementary material for: Streptomyces songxianensis sp. nov. SX92T: biocontrol of tobacco black shank and modulation of the rhizosphere microbiome
Source: Front Microbiol. 2026 Aug 25;17:1820235. doi: 10.3389/fmicb.2026.1820235 (PMC13550158; doi:10.3389/fmicb.2026.1820235)
Supplement: Supplementary file 5 [file Supplementary_file_5.docx]

**Table S5.** Composition and content of respiratory quinones in the cellular membrane of B-24802^T^

| Peak Number | Retention Time (min) | Peak Area (mAU·min) | Peak Height (mAU) | Relative Area (%) | Quinone Type |
| --- | --- | --- | --- | --- | --- |
| 1 | 30.02 | 515585 | 11325 | 11.64 | MK-9(H_4_) |
| 2 | 32.919 | 712443 | 13334 | 16.085 | MK-9(H_4_) |
| 3 | 34.411 | 556745 | 10197 | 12.57 | MK-9(H_6_) |
| 4 | 37.784 | 2094514 | 33523 | 47.288 | MK-9(H_8_) |
| 5 | 42.93 | 549955 | 8647 | 12.416 | MK-10(H_4_) |
