# Supplementary material for: Streptomyces songxianensis sp. nov. SX92T: biocontrol of tobacco black shank and modulation of the rhizosphere microbiome
Source: Front Microbiol. 2026 Aug 25;17:1820235. doi: 10.3389/fmicb.2026.1820235 (PMC13550158; doi:10.3389/fmicb.2026.1820235)
Supplement: Supplementary file 6 [file Supplementary_file_6.docx]

**Table S6.** Statistics of sequencing reads and assembly data from rhizosphere soil samples in the control and SX92^T^ treated groups

| Sample Name | Raw reads | Raw bases (bp) | Clean reads | Clean bases (bp) | contigs | Contigs bases (bp) | N50 (bp) | N90 (bp) | Max (bp) | Min (bp) |
| --- | --- | --- | --- | --- | --- | --- | --- | --- | --- | --- |
| CK-1 | 94,061,064 | 14,203,220,664 | 90,881,318 | 13,711,976,449 | 607,709 | 395,259,201 | 621 | 414 | 128359 | 300 |
| CK-2 | 80,916,012 | 12,218,317,812 | 77,704,986 | 11,724,176,798 | 519,914 | 355,430,763 | 670 | 421 | 52460 | 300 |
| CK-3 | 105,155,848 | 15,878,533,048 | 95,674,174 | 14,419,969,435 | 598,148 | 395,102,476 | 640 | 416 | 37391 | 300 |
| SX92^T^ -1 | 96,952,216 | 14,639,784,616 | 91,577,662 | 13,797,566,491 | 263,876 | 263,662,780 | 1158 | 435 | 614953 | 300 |
| SX92^T^ -2 | 95,354,356 | 14,398,507,756 | 86,850,710 | 13,082,474,238 | 388,050 | 279,747,398 | 661 | 416 | 329600 | 300 |
| SX92^T^ -3 | 124,361,228 | 18,778,545,428 | 121,082,608 | 18,256,695,933 | 174,347 | 256,278,310 | 4050 | 482 | 944060 | 300 |

Note: Sample names -1, -2, -3 are biological replicates.
